# Overview of Availability, Cost, and Affordability of Antibiotics for Adults in Jordan: An AWaRe Classification Perspective

**DOI:** 10.3390/antibiotics12111576

**Published:** 2023-10-29

**Authors:** Feras Darwish Elhajji, Sahar Abuhasheesh, Ahmed Al Rusasi, Mamoon A. Aldeyab

**Affiliations:** 1 Faculty of Pharmacy, Applied Science Private University, Amman 11937, Jordan; 2 School of Medicine, University of Jordan, Amman 11942, Jordan; 3 Jordan Pharmacists Association, Amman 11118, Jordan; 4 Department of Pharmacy, School of Applied Sciences, University of Huddersfield, Huddersfield HD1 3DH, UK; m.aldeyab@hud.ac.uk

**Keywords:** antibiotic, AWaRe classification, affordability, cost per DDD, access, watch, reserve, MAH, Jordan

## Abstract

Antimicrobial resistance (AMR) poses a global public health threat, with rates directly linked with consumption. The World Health Organization (WHO)’s AWaRe classification aims to guide antibiotic use, and is influenced by drug availability, affordability, and economic policies. In Jordan, a high proportion of consumed antibiotics belong to the ‘Watch’ category. Data from the WHO’s AWaRe classification, the Essential Medicines List, and the Jordan Food and Drug Administration were analyzed. Antibiotics for adults were classified, their costs per defined daily dose (DDD) were determined and their affordability assessed. In 2023, 43 injectable and 43 oral antibiotics for adults were registered in Jordan. ‘Watch’ antibiotics were the most common. ‘Access’ antibiotics had the lowest cost/DDD. ‘Reserve’ antibiotics were the most expensive, with few generics. Injectable antibiotics had a negative correlation between cost and the number of alternatives. Affordability was higher for oral antibiotics compared with injectable ones. ‘Reserve’ antibiotics were generally unaffordable. This study highlights the need to promote ‘Access’ antibiotics over other categories by encouraging the registration of missing ‘Access’ antibiotics and adjusting the prices of ‘Watch’ and ‘Reserve’ antibiotics. Competition among generics can lead to lower prices, increasing affordability and accessibility. We emphasize the importance of the AWaRe classification in guiding antibiotic use in Jordan.

## 1. Introduction

Antimicrobial resistance (AMR), as a seriously identified global public health problem, is becoming an urgent and demanding issue [1,2,3]. It has been proved that rates of AMR are directly related to rates of antimicrobial consumption. The frequent and irrational use of broad-spectrum antibiotics is a main cause of this problem. In addition to its burden on health, AMR exerts an economic pressure that requires searching for new, expensive antibiotics to treat resistant bacterial infections [2].

World Health Organization (WHO) experts have created a classification of antibiotics that is expected to slow down the resistance rates against available antibiotics. This classification is based on the listing of antibiotics into three groups, i.e., ‘Access’, ‘Watch’, and ‘Reserve’ antibiotics (AWaRe). By considering AWaRe classification during antibiotics prescribing practice and antimicrobial stewardship efforts, this tool can help in properly selecting antibiotics with a lesser possibility of causing antibiotic resistance. In other words, considering the AWaRe classification of antibiotics may rationalize antibiotic consumption [4,5]. The WHO issued a target that at least 60% of antibiotic consumption are from agents belonging to the ‘Access’ group [4,6], however, 70.5% of the prescribed antibiotics issued in Jordanian hospitals have belonged to the ‘Watch’ category [7].

Antimicrobial stewardship that includes all related dimensions can provide solutions for the fight against AMR [3]. However, there are different factors which may affect the quality of antibiotic prescription. One of the factors that may lead to the increased prescribing of broad-spectrum antibiotics is the shortage in primary healthcare settings [8]. Meanwhile, economic policies, such as those associated with the implementation of income-based pharmaceutical co-payments, were found to reduce consumption of antibiotics in general [9,10]. At the same time, low public prices of antibiotics can enhance their affordability and consumption [11].

Between 1990 and 2010, development of new antibiotics by large pharmaceutical companies was seriously reduced. In fact, the number of companies researching antibiotics dropped from 18 to 4. At the same time, the number of newly approved antibiotics had been affected to such an extent that it reached its low point even as worrying levels of resistance to available antibiotics were reached [12]. Many guidelines recommend the use of older antibiotics whenever appropriate. Regardless of their safety and efficacy, the availability of the older antibiotics is a challenge from a commercial point of view [13].

Affordability of a drug or treatment can be defined as a measurement of the ease or feasibility of the members of a society to pay for that drug or treatment. It is correlated with drug prices, insurance coverage, a family’s financial status, and, sometimes, the indication of the drug [14]. In 2008, the WHO and Health Action International (HAI) defined treatment affordability based on the daily wage of the lowest-paid unskilled government worker [15]. The poor access to antibiotics and their insufficient affordability in low- and middle-income countries in the world is expected to worsen the AMR problem due to the inability of people in those countries to afford full treatment for their infections [2,3]. This may be considered a reflection of the inverse relationship between the cost of treatment and the consumption of drugs.

Ideally, ‘Access’ antibiotics should have an affordable cost, as to enhance their use as first or second lines of treatment [5,16]. Discovering that ‘Access’ antibiotics are more affordable than other classes, or that ‘Reserve’ antibiotics have higher prices, may aid in the achievement of reaching a higher relative consumption of ‘Access’ antibiotics while reserving the ‘Reserve’ antibiotics for limited serious cases of infection.

Broad-spectrum antibiotics are more commonly available in injection rather than in oral dosage forms. Injectable antibiotics have been most commonly administered in hospital settings in west and central Asia, Latin America, and eastern and southern Europe. Jordan is a country located in west Asia, where broad-spectrum parenteral antibiotics, such as third-generation cephalosporins, are frequently prescribed for both treatment and prophylaxis in hospitals. Versporten et al. have stated that, in 2015, more than 80% of inpatients in these areas, including Jordan, had been administered broad-spectrum antibiotics [17].

This study aimed to explore registered antibiotics for adults in Jordan according to their AWaRe classification, and to determine the relationship between cost, availability and affordability, and how the antibiotic was classified.

## 2. Results

**Figure 1:**
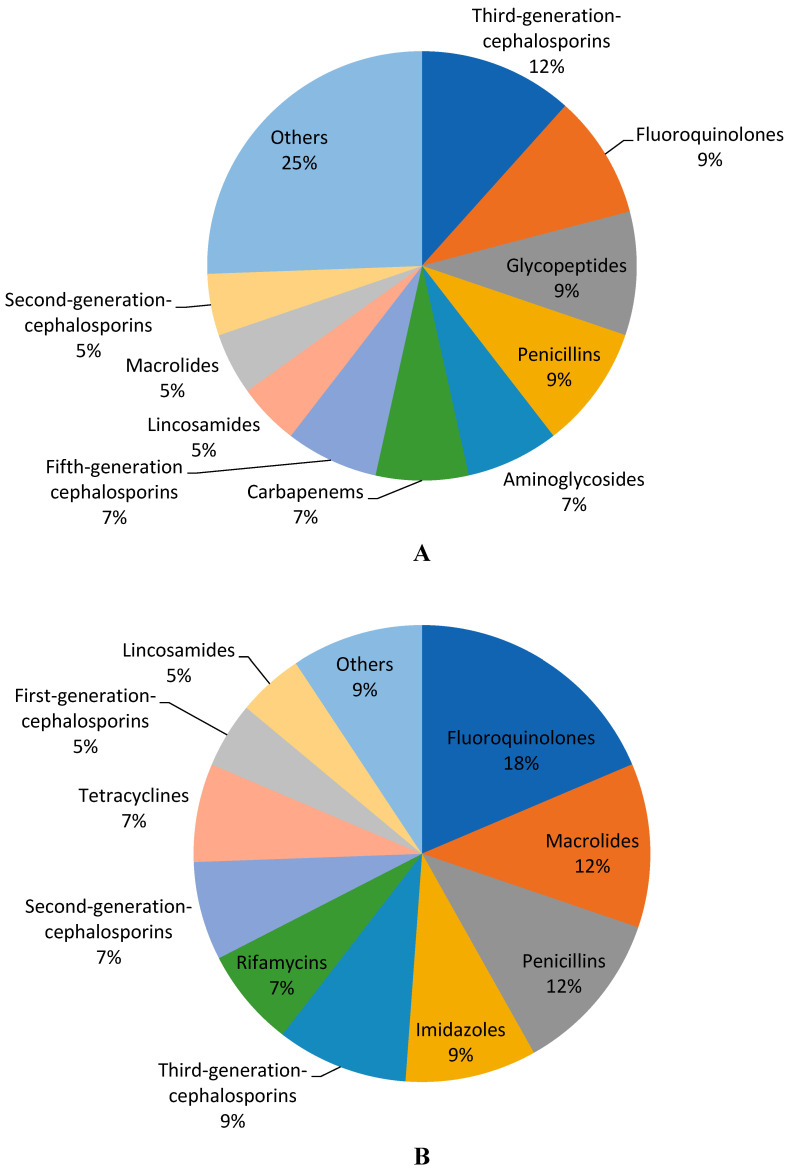
Percentages of the types of antibiotics according to pharmacological class, (**A**) injection and (**B**) oral.

Until the end of August 2023, the total number of injectable antibiotics for adults that had been registered in Jordan by the Jordan Food and Drug Administration (JFDA) was 43, and was also 43 for oral antibiotics. The injectable and oral antibiotics for adults in Jordan, along with their WHO AWaRe groups, listing status on the Essential Medicines List (EML) in 2023, their defined daily dose (DDD), mean cost of the DDD, and number of marketing authorization holders (MAH), are listed in Table 1 and Table 2. Injectable and oral antibiotics for adults in Jordan belong to various pharmacological classes. About 40% of the types of injectable antibiotics were third-generation cephalosporins (12%), fluoroquinolones (9%), glycopeptides (9%), or penicillins (9%). At the same time, fluoroquinolone antibiotics represented the majority of the oral types of antibiotics (18%), followed by macrolides and penicillins, with a 12% share for each (Figure 1).

About half of the injectable antibiotics for adults in Jordan (48.8%) belonged to the ‘Watch’ group. ‘Access’ and ‘Reserve’ groups represented 27.9% and 23.3%, respectively. All of the ‘Access’ injectable antibiotics were listed on the EML. The majority of ‘Reserve’ injectable antibiotics (9 out of 10) had only one MAH in the Jordanian market.

Gentamycin, an ‘Access’ injectable aminoglycoside, had a mean public cost per DDD equal to 2.20 Jordanian Dinar (JOD)/DDD. It was found to have the lowest cost DDD of the injectable antibiotics. It had three alternative brands in the market and was listed on the EML. The antibiotic with the highest mean public cost per DDD was oritavancin (609.93 JOD/DDD). Oritavancin is a ‘Reserve’ glycopeptide, with only the originator drug, not listed on the EML, available in Jordan.

Similar to injectable antibiotics, the majority of the oral antibiotics for adults belonged to the ‘Watch’ group, which represented more than 60% of the total oral antibiotics, followed by ‘Access’ antibiotics (37.3%). Linezolid was the only oral antibiotic for adults in Jordan that belongs to ‘Reserve’ group. It was listed on the EML and had a total of four originator and generic alternatives. Most of the ‘Access’ oral antibiotics were listed in the EML. At the same time, only about 30% of oral ‘Watch’ antibiotics could be found in EML.

Doxycycline, an ‘Access’ oral tetracycline, had a mean public cost per DDD equal to 0.24 JOD/DDD. It was found to have the lowest cost of DDD out of the oral antibiotics. It had eight alternative brands in Jordan and was listed in the EML. The antibiotic with the highest mean public cost per DDD was delafloxacin (66.66 JOD/DDD). Delafloxacin is a ‘Watch’ fluoroquinolone for which only the originator is available in Jordan, though it is not listed in the EML.

The injectable antibiotic with the highest number of alternatives (originator and generics) was ceftriaxone. Ceftriaxone, a ‘Watch’ group third-generation cephalosporin had 11 MAH in Jordan. It also had the highest cost ratio and % of cost variation, i.e., 6.78 and 577.9%, respectively (Table 3). On the other hand, ciprofloxacin was the oral antibiotic with the highest number of alternatives. This ‘Watch’-classified fluoroquinolone had 17 MAHs in Jordan. However, oral amoxicillin had a higher cost ratio (5.92) and % cost variation (492.3%) (Table 4).

The means of the costs of DDDs for all registered injectable and oral adult antibiotics in Jordan according to their mean prices for public were notably different according to their AWaRe classification. The difference in costs of DDDs was statistically significant between AWaRe groups (Kruskal–Wallis test) (Table 5). More specifically, the difference in mean cost was statistically significant between the ‘Access’ and ‘Watch’ antibiotics on one side and the ‘Reserve’ antibiotics on the other (ρ < 0.001), according to post hoc Tukey HSD. While the mean cost/DDD for the ‘Reserve’ antibiotics exceeded 150 JOD, mean cost/DDD for the ‘Watch’ and ‘Access’ antibiotics were around 13 JOD and 3 JOD, respectively.

AWaRe groups of antibiotics were also variable regarding number of MAHs. ‘Watch’ injectable and oral antibiotics had the highest mean number of MAHs (4.74), while mean MAH for the ‘Reserve’ antibiotics was 1.36. The difference was statistically significant (ρ = 0.003).

The costs of DDDs of adult injectable antibiotics in Jordan were found to inversely correlate with the number of MAHs (i.e., available number of alternatives registered by the JFDA). Spearman’s rho correlation coefficient was –0.354, and was statistically significant (ρ = 0.020). A similar correlation could not be found upon comparing the costs of DDDs of oral antibiotics with the number of available brands. Although they had an inverse relationship similar to the injectable antibiotics, Spearman’s rho correlation was not statistically significant for the relationship between the oral cost of DDDs and the number of MAHs.

### Affordability of the Antibiotics

**Figure 2:**
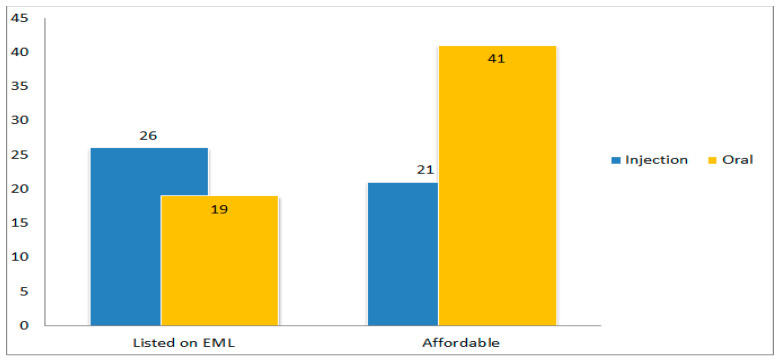
Number of injectable and oral antibiotics according to listing on the EML and affordability (EML: essential medicines list).

A total of 26 injectable antibiotic (60.5%) and 19 oral antibiotic (44.2%) were listed in the EML. However, the difference in percentage of listing in the EML between injectable and oral antibiotics was not found to be statistically significant. At the same time, oral antibiotics were significantly more affordable (ρ < 0.001) than injectable antibiotics (Figure 2).

Of those oral antibiotics listed on the EML, the ‘Reserve’ antibiotic linezolid was the only antibiotic considered to be non-affordable. The non-affordable injectable antibiotics that were listed on the EML were four ‘Watch’ antibiotics (cefotaxime, imipenam with cilastatin, meropenem, and piperacillin with tazobactam) and four ‘Reserve’ antibiotics (ceftazidime with avibactam, ceftolozane with tazobactam, colistin, and linezolid).

All injectable and oral antibiotics that belong to the ‘Access’ group were found to be affordable. On the other hand, all of the ‘’Reserve’ group antibiotics for both routes of administration were found to be not affordable. Delafloxacin was the only oral ‘Watch’ antibiotic to be considered not affordable. on the contrary, approximately half of the ‘Watch’ injectable antibiotics (11 antibiotics) were considered affordable. The difference in affordability between all of the adult antibiotics in Jordan according to the AWaRe classification was statistically significant. Affordability ranged from 100% for the ‘Access’ antibiotics to 0% for the ‘Reserve’ antibiotics, with ~75% affordability among the ‘Watch’ antibiotics (Table 5).

By comparing the number of MAHs for all of the antibiotics according to their affordability classification, 64 antibiotics were found to be affordable, with a 4.69 mean number of MAHs (alternatives). The other 22 non-affordable antibiotics had a mean number of MAHs equal to 1.95. The difference in MAHs was statistically significant (ρ = 0.001) (Table 6).

The injectable and oral antibiotics had a mean cost ratio of approximately 1.9. The mean % cost variation was 92.4 for the injectable and 97.3 for the oral antibiotics. Neither the cost ratio nor the % cost variation were significantly different.

## 3. Discussion

This research aimed to draw a picture of the availability of antibiotics for adults in Jordan, with brief cost and regulatory comparisons. All of the included data were extracted from the WHO sources or the JFDA, which is the sole governmental drug regulatory body in Jordan.

In addition to the AWaRe classification, the route of administration of the antibiotic was considered a primary classification for the antibiotics. The antibiotics for adults can be given to or administered by patients in different dosage forms via different routes according to the settings, indication, and severity of the condition. Sometimes, injectable and oral antibiotics can be notably different in DDDs and cost.

An extensive literature search was conducted to try to find published studies or statistics about prescription or consumption of antibiotics by AWaRe groups in Jordan. There was a shortage of published statistics about the consumption of antibiotics according to AWaRe classification in Jordan at larger scales, including the national scale. An interventional study that was published in 2023 had used a single hospital’s antibiotic prescription data grouped according to AWaRe classification. It calculated antibiotics DDDs for adults and pediatrics that had been dispensed by the hospital [18]. Al-Azzam et al. used the JFDA-derived annual national antimicrobial consumption for 2019 and 2020 to assess the impact of COVID-19 on national antimicrobial consumption in Jordan. ‘Watch’ antibiotics faced an increase in consumption in 2020. Azithromycin showed a great leap in prescription rates during the pandemic [19]. In conclusion, this study shows that azithromycin as a ‘Watch’ antibiotic for adults had 16 MAHs for oral azithromycin and 3 MAHs for the injection, which makes it one of the more highly available antibiotics in Jordan.

A considerable number of antibiotics for adults, whether formulated to be taken orally or parenterally, were not found to be registered in Jordan. Many of the ‘missing’ antibiotics from the Jordanian market can be described as older antibiotics that belong to the ‘Access’ group. Regardless of the benefits of their use, older antibiotics lack active marketing and will have relatively low prices and high registration and re-registration fees so that, as a result, MAHs might decide not to keep them available [13].

A great proportion of the registered antibiotics in Jordan belonged to third-generation cephalosporins, fluoroquinolones, and penicillins. Third-generation cephalosporins and fluoroquinolones, in addition to penicillins with β-lactamase inhibitors as pharmacological classes, were among the highest prescribed antibiotics in hospitals in Jordan and worldwide [7,17,20,21]. The route of administration, e.g., parenteral or oral, can be considered an important variable in studies that are concerned about exploring the availability and affordability of antibiotics. In fact, in 2015, more than 80% of hospital inpatients in Jordan and other countries were administered broad-spectrum antibiotics, such as third-generation cephalosporins, which are mainly injectable ‘Watch’ antibiotics, for treatment or surgery prophylaxis purposes [17]. Ceftriaxone, being a third-generation cephalosporin, seems to be the most available injectable antibiotic in Jordan. It had the highest number of alternatives, which led to a wide cost ratio and cost % variation.

The reported consumption of ‘Access’ antibiotics in 28 European countries in 2021 was approximately 60% of the total consumed antibiotics in both the community and hospital sectors [6]. Our study shows that the majority of registered antibiotics for adults in Jordan belonged to the ‘Watch’ group. At the same time, the number of MAHs, representing available originators and generics, was the highest among the ‘Watch’ group. Published results of surveillance on antibiotic consumption in 2015 showed that about 60% of the consumed DDDs of antibiotics per 1000 inhabitants per day in Jordan belonged to the ‘Watch’ group [2]. This is consistent with the global analysis of pharmaceutical sales data between 2000 and 2015 [16]. It seems that more actions, such as reviewing and implementing the policies related to antibiotic prescription practices and regulatory and drug pricing guidelines, should be taken to enhance the prescription of the ‘Access’ group of antibiotics over other antibiotics.

We found that the registered ‘Reserve’ group of antibiotics in Jordan had the highest mean cost/DDD and the lowest mean number of MAHs (originators and generics). The least expensive oral and injectable DDDs of antibiotics (i.e., doxycycline and gentamycin, respectively) belong to the ‘Access’ group. Generally, the low price of a drug can make it more affordable [11]. Cost-effectiveness of the drugs, including antibiotics, can partially be attained by enhancing competition between available treatments. This competition can be sustained by enabling the availability for prescribers of more generic versions of the antibiotics, which will in turn eventually decrease prices and cost [12]. It must be noted that generics are priced by the JFDA and their price never exceeds 70% of the new drug or originator’s price. At the same time, the availability of therapeutic alternatives may enhance supply and decrease tender prices.

None of the ‘Reserve’ antibiotics are considered affordable in Jordan, in contrary with the ‘Access’ antibiotics. Such a finding is in line with the target of keeping ‘Access’ antibiotics more affordable while leaving ‘Reserve’ antibiotics as a last resort choice [5,16].

This study has limitations that should be addressed. The data that were investigated lacked correlation with real-time antibiotic consumption figures. However, the conducted research can still be conclusive. Restricting the number of available alternatives of the ‘Reserve’ antibiotics could ensure higher prices and reduced affordability and, as a result, hopefully limit consumption and lower resistance rates in the future. Classifying certain ‘Watch’ antibiotics to be ‘Reserve’ can also be suggested. Moreover, the concept of affordability, in terms of the way it was judged, was not the optimal measure by which to reflect the actual affordability of antibiotics. Jordan has a diverse healthcare system with variable health insurance coverage packages. Linking the affordability of antibiotics with their cost/DDD in relation to the minimum daily wage helped in defining a threshold for the cost, above which the DDD would be expensive.

## 4. Methods

### 4.1. Sources of Data

The data that were collected to conduct this study were extracted from four electronic resources that were available online. The resources were:

1- The WHO AWaRe classification of antibiotics for the evaluation and monitoring of use, 2023. This was downloaded from the 2023 AWaRe classification webpage of the WHO [4]. Information such as the 2023 AWaRe classification of antibiotics, pharmacological class, and the Anatomical Therapeutic Chemical (ATC) code of the antibiotics were extracted from this source.
2- The 23rd EML that was issued in 2023 [22] was utilized to determine the status of listing of each antibiotic (according to its dosage form and route of administration).
3- An index that uses ATC codes was used to find each antibiotic’s DDD. The ATC/DDD index is a searchable version of the complete ATC index with DDDs provided online by the WHO Collaborating Centre for Drug Statistics Methodology and the Norwegian Institute of Public Health [23].
4- Drug information, prices, and leaflets webpage by JFDA [24]. Prices of registered antibiotic items in all available packs for all marketing authorization holders in Jordan could be found through this official webpage.

Each antibiotic mentioned and classified in the WHO AWaRe classification list was searched for its registration in Jordan via the JFDA website. Antibiotics that were included in the study were the antibiotics registered and priced in Jordan in a dosage form for adults (i.e., vial or ampule for injection, or oral tablet or capsule) to be given parenterally or orally.

### 4.2. Cost of DDD

The WHO have defined DDD as “the assumed average maintenance dose per day for a drug used for its main indication in adults” [25]. The route of administration of the antibiotic for adult was considered for determination of the DDD. The DDDs for each antibiotic, given either parenterally or orally, was directly matched with the ATC code of that specific antibiotic. To calculate the cost of DDD as JOD per DDD (JOD/DDD), the equation was required to have the public price of the antibiotic as a drug item, its pack size (i.e., number of units in the pack), strength of the antibiotic, and its DDD. The mean cost per DDD was then calculated for each antibiotic. The highest and lowest costs per DDD were also collected.

### 4.3. Affordability

The highest and lowest cost/DDD for each antibiotic were used to calculate cost ratio and percentage (%) of cost variation. The cost ratio was calculated by dividing the highest cost/DDD on the lowest cost/DDD. The % cost variation was calculated as the percentage difference of the highest and lowest cost/DDD to the lowest cost/DDD [26]. Antibiotic affordability was calculated according to the WHO and HAI definitions [15,26]. The Jordanian Ministry of Labor had set the minimum monthly wage for the years 2023 and 2024 at 260 JOD [27]. The threshold of antibiotic affordability was set to be the daily wage that had been set by the government. If the lowest cost/DDD of each antibiotic was less than the minimum daily wage (~8.67 JOD), it would have been considered ‘affordable’.

In this study, the MAH in Jordan was considered to be the pharmaceutical company or drug store that holds the registration rights and is responsible for the regulatory affairs related to the antibiotic drug. The MAH can register an originator with more than one generic of the same antibiotic, and each type of antibiotic can be available in different dosage forms (e.g., oral tablets and capsules), different strengths, and different pack size. The number of MAHs is a metric that aims to represent a scale of alternatives to a registered antibiotic, and, indirectly, its availability.

### 4.4. Statistical Analysis

All data were transferred to IBM SPSS^®^ Statistics 24 and underwent descriptive analysis. Pearson’s chi-squared test was used for categorical comparison, based specifically on a drug’s listing in the EML and its affordability. Nonparametric tests were conducted because of the nature of the data. The Kruskal–Wallis test was used to determine the difference in the mean cost of DDDs and the mean number of MAHs according to AWaRe classification. Further determination of the statistical significance of the difference between the categories was undertaken via ANOVA post hoc Tukey HSD. The Mann–Whitney U test was used to determine the difference in mean cost ration and % cost variation according to the route of administration, and the difference in mean number of MAHs according to affordability. For the correlation between costs of the DDDs and the number of MAHs, the test was Spearman’s correlation.

## 5. Conclusions

In conclusion, a relationship was confirmed between the AWaRe classification of the antibiotic and its cost, availability, and affordability parameters. Availability and affordability of the antibiotic can be enhanced by having a lower price and a higher number of generics. Jordan has more available and affordable ‘Access’ antibiotics for adults than ‘Reserve’ antibiotics. Efforts should be made to enhance antimicrobial stewardship, regulate the dispensing of antibiotics, encourage the registration of the missing antibiotics, grant privileges to the registration of more generics, and increase prices of the ‘Watch’ and ‘Reserve’ antibiotics.

## Figures and Tables

**Table 1:** Injectable antibiotics and their classification and cost.

WHO AWaRe Category	Antibiotic	ATC Code	Listed on EML	DDD (g)	Mean Cost (JOD/DDD)	Number of MAH
Access	Amikacin	J01GB06	Yes	1	7.66	4
	Amoxicillin/clavulanic acid	J01CR02	Yes	3	4.45	3
	Ampicillin	J01CA01	Yes	6	3.9	2
	Benzathine-benzylpenicillin	J01CE08	Yes	3.6	4.67	1
	Cefazolin	J01DB04	Yes	3	5.76	2
	Clindamycin	J01FF01	Yes	1.8	14.89	3
	Cloxacillin	J01CF02	Yes	2	4.65	1
	Gentamicin	J01GB03	Yes	0.24	2.2	3
	Metronidazole	J01XD01	Yes	1.5	4.75	8
	Procaine-benzylpenicillin	J01CE09	Yes	0.6	2.54	1
	Spectinomycin	J01XX04	Yes	3	5.93	1
	Sulfamethoxazole/trimethoprim	J01EE01	Yes	2	7.24	1
Watch	Azithromycin	J01FA10	No	0.5	8.03	3
	Cefepime	J01DE01	No	4	20.74	3
	Cefotaxime	J01DD01	Yes	4	21.15	3
	Cefoxitin	J01DC01	No	6	35.76	1
	Ceftazidime	J01DD02	Yes	4	11.59	6
	Ceftizoxime	J01DD07	No	4	33.58	2
	Ceftriaxone	J01DD04	Yes	2	11.31	11
	Cefuroxime	J01DC02	Yes	3	5.92	7
	Ciprofloxacin	J01MA02	Yes	0.8	30.61	7
	Clarithromycin	J01FA09	Yes	1	27.1	1
	Delafloxacin	J01MA23	No	0.6	111.28	1
	Ertapenem	J01DH03	No	1	24.18	2
	Imipenem/cilastatin	J01DH51	Yes	2	31.91	5
	Levofloxacin	J01MA12	No	0.5	13.94	6
	Lincomycin	J01FF02	No	1.8	5.34	5
	Meropenem	J01DH02	Yes	3	31.33	5
	Moxifloxacin	J01MA14	No	0.4	16.15	3
	Piperacillin/tazobactam	J01CR05	Yes	14	30.75	4
	Teicoplanin	J01XA02	No	0.4	7.73	5
	Tobramycin	J01GB01	No	0.24	12.69	1
	Vancomycin	J01XA01	Yes	2	18.74	9
Reserve	Ceftaroline-fosamil	J01DI02	No	1.2	72.47	1
	Ceftazidime/avibactam	J01DD52	Yes	6	268.38	1
	Ceftobiprole-medocaril	J01DI01	No	1.5	133.9	1
	Ceftolozane/tazobactam	J01DI54	Yes	3	221.96	1
	Colistin	J01XB01	Yes	9	63.03	1
	Daptomycin	J01XX09	No	0.28	49.06	1
	Linezolid	J01XX08	Yes	1.2	64.45	1
	Oritavancin	J01XA05	No	1.2	609.93	1
	Telavancin	J01XA03	No	0.75	93.6	1
	Tigecycline	J01AA12	No	0.1	60.58	2

ATC: Anatomical Therapeutic Chemical; EML: Essential Medicines List; JOD: Jordanian Dinar; DDD: defined daily dose; MAH: marketing authorization holder.

**Table 2:** Oral antibiotics and their classifications and cost.

WHO AWaRe Category	Antibiotic	ATC Code	Listed on EML 2023	DDD (g)	Mean Cost (JOD/DDD)	Number of MAH
Access	Amoxicillin	J01CA04	Yes	1.5	0.46	12
	Amoxicillin/clavulanic acid	J01CR02	Yes	1.5	0.83	10
	Ampicillin	J01CA01	No	2	0.46	4
	Cefadroxil	J01DB05	No	2	1.37	6
	Cefalexin	J01DB01	Yes	2	0.78	9
	Clindamycin	J01FF01	Yes	1.2	1.66	5
	Cloxacillin	J01CF02	Yes	2	0.78	2
	Doxycycline	J01AA02	Yes	0.1	0.24	8
	Flucloxacillin	J01CF05	Yes	2	0.83	1
	Metronidazole	P01AB01	Yes	2	0.34	7
	Ornidazole	P01AB03	No	1.5	1.76	2
	Phenoxymethylpenicillin	J01CE02	Yes	2	0.39	1
	Secnidazole	P01AB07	No	2	3.38	1
	Sulfamethoxazole/trimethoprim	J01EE01	Yes	2	0.37	3
	Tetracycline	J01AA07	No	1	0.24	1
	Tinidazole	P01AB02	No	2	2.28	3
Watch	Azithromycin	J01FA10	Yes	0.3	0.72	16
	Cefaclor	J01DC04	No	1	1.19	8
	Cefdinir	J01DD15	No	0.6	3.72	4
	Cefditoren-pivoxil	J01DD16	No	0.4	1.97	1
	Cefixime	J01DD08	Yes	0.4	2.41	10
	Cefpodoxime-proxetil	J01DD13	No	0.4	2.9	5
	Cefprozil	J01DC10	No	1	3.25	2
	Cefuroxime	J01DC02	No	0.5	0.59	10
	Ciprofloxacin	J01MA02	Yes	1	1.15	17
	Clarithromycin	J01FA09	Yes	0.5	0.87	11
	Delafloxacin	J01MA23	No	0.9	66.66	1
	Erythromycin	J01FA01	No	2	0.58	2
	Levofloxacin	J01MA12	Yes	0.5	1.46	14
	Lincomycin	J01FF02	No	1.8	0.85	1
	Lomefloxacin	J01MA07	No	0.4	1.29	1
	Moxifloxacin	J01MA14	Yes	0.4	1.77	8
	Norfloxacin	J01MA06	No	0.8	0.58	4
	Ofloxacin	J01MA01	No	0.4	1.54	1
	Pefloxacin	J01MA03	No	0.8	1.09	2
	Rifabutin	J04AB04	Yes	0.15	2.91	1
	Rifampicin	J04AB02	Yes	0.6	0.43	2
	Rifaximin	A07AA11	No	0.6	1.57	1
	Roxithromycin	J01FA06	No	0.3	0.76	5
	Spiramycin	J01FA02	No	3	0.66	3
	Fosfomycin	J01XX01	No	3	4.48	2
	Minocycline	J01AA08	No	0.2	0.85	1
Reserve	Linezolid	J01XX08	Yes	1.2	45.98	4

ATC: Anatomical Therapeutic Chemical; EML: Essential Medicines List; JOD: Jordanian Dinar; DDD: defined daily dose; MAH: marketing authorization holder.

**Table 3:** Cost ratio and cost variation of injectable antibiotics in Jordan.

WHO AWaRe Category	Antibiotic	Cost Range (JOD/DDD)	Cost Ratio	% Cost Variation	Affordable
Access	Amikacin	6.15–8.83	1.44	43.6	Yes
	Amoxicillin/clavulanic acid	3.27–5.58	1.71	70.6	Yes
	Ampicillin	2.99–4.91	1.64	64.2	Yes
	Benzathine-benzylpenicillin	4.67	1.00	0.0	Yes
	Cefazolin	4.70–6.48	1.38	37.9	Yes
	Clindamycin	6.09–19.20	3.15	215.3	Yes
	Cloxacillin	4.03–5.27	1.31	30.8	Yes
	Gentamicin	1.11–3.78	3.41	240.5	Yes
	Metronidazole	3.26–9.14	2.80	180.4	Yes
	Procaine-benzylpenicillin	2.54	1.00	0.0	Yes
	Spectinomycin	5.93	1.00	0.0	Yes
	Sulfamethoxazole/trimethoprim	7.24	1.00	0.0	Yes
Watch	Azithromycin	5.24–10.62	2.03	102.7	Yes
	Cefepime	14.64–25.40	1.73	73.5	No
	Cefotaxime	11.92–24.24	2.03	103.4	No
	Cefoxitin	35.76	1.00	0.0	No
	Ceftazidime	6.88–18.56	2.70	169.8	Yes
	Ceftizoxime	22.90–37.44	1.63	63.5	No
	Ceftriaxone	2.98–20.20	6.78	577.9	Yes
	Cefuroxime	3.39–10.12	2.99	198.5	Yes
	Ciprofloxacin	11.96–56.34	4.71	371.1	No
	Clarithromycin	27.10	1.00	0.0	No
	Delafloxacin	111.28	1.00	0.0	No
	Ertapenem	18.69–29.66	1.59	58.7	No
	Imipenem/cilastatin	25.00–39.68	1.59	58.7	No
	Levofloxacin	7.30–22.94	3.14	214.2	Yes
	Lincomycin	4.68–6.66	1.42	42.3	Yes
	Meropenem	16.69–57.11	3.42	242.2	No
	Moxifloxacin	7.20–22.19	3.08	208.2	Yes
	Piperacillin/tazobactam	25.13–36.69	1.46	46.0	No
	Teicoplanin	3.69–12.40	3.36	236.0	Yes
	Tobramycin	12.69	1.00	0.0	No
	Vancomycin	8.01–29.28	3.66	265.5	Yes
Reserve	Ceftaroline-fosamil	72.47	1.00	0.0	No
	Ceftazidime/avibactam	268.38	1.00	0.0	No
	Ceftobiprole-medocaril	133.90	1.00	0.0	No
	Ceftolozane/tazobactam	221.96	1.00	0.0	No
	Colistin	59.72–66.33	1.11	11.1	No
	Daptomycin	49.06	1.00	0.0	No
	Linezolid	61.06–67.84	1.11	11.1	No
	Oritavancin	609.93	1.00	0.0	No
	Telavancin	93.60	1.00	0.0	No
	Tigecycline	51.66–69.50	1.35	34.5	No

JOD: Jordanian Dinar; DDD: defined daily dose.

**Table 4:** Cost ratio and cost variation of oral antibiotics in Jordan.

WHO AWaRe Category	Antibiotic	Cost Range (JOD/DDD)	Cost Ratio	% Cost Variation	Affordable
Access	Amoxicillin	0.13–0.77	5.92	492.3	Yes
	Amoxicillin/clavulanic acid	0.61–1.29	2.11	111.5	Yes
	Ampicillin	0.30–0.79	2.63	163.3	Yes
	Cefadroxil	0.85–2.48	2.92	191.8	Yes
	Cefalexin	0.29–1.22	4.21	320.7	Yes
	Clindamycin	1.38–2.00	1.45	44.9	Yes
	Cloxacillin	0.67–0.83	1.24	23.9	Yes
	Doxycycline	0.17–0.35	2.06	105.9	Yes
	Flucloxacillin	0.83	1.00	0.0	Yes
	Metronidazole	0.19–0.69	3.63	263.2	Yes
	Ornidazole	1.56–1.95	1.25	25.0	Yes
	Phenoxymethylpenicillin	0.39	1.00	0.0	Yes
	Secnidazole	3.01–3.75	1.25	24.6	Yes
	Sulfamethoxazole/trimethoprim	0.21–0.63	3.00	200.0	Yes
	Tetracycline	0.21–0.26	1.24	23.8	Yes
	Tinidazole	2.09–2.78	1.33	33.0	Yes
Watch	Azithromycin	0.47–0.90	1.91	91.5	Yes
	Cefaclor	0.78–1.43	1.83	83.3	Yes
	Cefdinir	3.40–4.47	1.31	31.5	Yes
	Cefditoren-pivoxil	1.97	1.00	0.0	Yes
	Cefixime	1.19–3.12	2.62	162.2	Yes
	Cefpodoxime-proxetil	2.30–3.83	1.67	66.5	Yes
	Cefprozil	3.00–3.66	1.22	22.0	Yes
	Cefuroxime	0.38–0.78	2.05	105.3	Yes
	Ciprofloxacin	0.45–2.38	5.29	428.9	Yes
	Clarithromycin	0.55–1.32	2.40	140.0	Yes
	Delafloxacin	66.66	1.00	0.0	No
	Erythromycin	0.31–0.80	2.58	158.1	Yes
	Levofloxacin	0.62–1.81	2.92	191.9	Yes
	Lincomycin	0.75–0.94	1.25	25.3	Yes
	Lomefloxacin	1.14	1.26	26.3	Yes
	Moxifloxacin	1.01–2.17	2.15	114.9	Yes
	Norfloxacin	0.50–0.73	1.46	46.0	Yes
	Ofloxacin	1.41–1.66	1.18	17.7	Yes
	Pefloxacin	1.03–1.13	1.10	9.7	Yes
	Rifabutin	2.91	1.00	0.0	Yes
	Rifampicin	0.41–0.47	1.15	14.6	Yes
	Rifaximin	1.57	1.00	0.0	Yes
	Roxithromycin	0.67–0.83	1.24	23.9	Yes
	Spiramycin	0.380.96	2.53	152.6	Yes
	Fosfomycin	3.78–5.17	1.37	36.8	Yes
	Minocycline	0.73–0.96	1.32	31.5	Yes
Reserve	Linezolid	29.70–82.50	2.78	177.8	No

JOD: Jordanian Dinar; DDD: defined daily dose.

**Table 5:** Costs of antibiotics (JOD/DDD) and number of MAHs according to AWaRe classification.

	Access	Watch	Reserve	Sig. (ρ)
N	28	47	11	
Mean JOD/DDD (S.E.)	3.03 (0.61)	13.11 (2.94)	153.03 (50.81)	<0.001 *
Mean MAHs (S.E.)	3.75 (0.60)	4.74 (0.60)	1.36 (0.28)	0.003 *
Affordable (%)	28 (100.0%)	34 (72.3%)	0 (0.0%)	<0.001 **

* Statistically significant according to Kruskal–Wallis test. ** Statistically significant according to Pearson’s chi-squared test.

**Table 6:** Number of MAHs according to the affordability of the antibiotics.

	Affordable	Non-Affordable	Sig. (ρ)
N	62	24	
Mean MAH (S.E.)	4.69 (0.50)	1.95 (0.30)	0.001 *

* Statistically significant according to the Mann–Whitney U test.

## Data Availability

The data are contained in the article.

## References

[1] Minarini L.A.D.R., de Andrade L.N., De Gregorio E., Grosso F., Naas T., Zarrilli R., Camargo I.L.B.C. (2020). Editorial: Antimicrobial Resistance as a Global Public Health Problem: How Can We Address It?. Front. Public Health.

[2] World Health Organization (2018). WHO Report on Surveillance of Antibiotic Consumption: 2016–2018 Early Implementation. Geneva. https://www.who.int/publications/i/item/who-report-on-surveillance-of-antibiotic-consumption.

[3] Cox J.A., Vlieghe E., Mendelson M., Wertheim H., Ndegwa L., Villegas M.V., Gould I., Hara G.L. (2017). Antibiotic stewardship in low- and middle-income countries: The same but different?. Clin. Microbiol. Infect..

[4] World Health Organization WHO AWaRe (access, watch, reserve) classification of antibiotics for evaluation and monitoring of use, 2023. Proceedings of the Selection and Use of Essential Medicines 2023: Executive Summary of the Report of the 24th WHO Expert Committee on the Selection and Use of Essential Medicines.

[5] (2022). The WHO AWaRe (Access, Watch, Reserve) Antibiotic Book.

[6] European Centre for Disease Prevention and Control (2022). Antimicrobial Consumption in the EU/EEA (ESAC-Net)—Annual Ep-idemiological Report 2021.

[7] Talaat M., Tolba S., Abdou E., Sarhan M., Gomaa M., Hutin Y.J.-F. (2022). Over-Prescription and Overuse of Antimicrobials in the Eastern Mediterranean Region: The Urgent Need for Antimicrobial Stewardship Programs with Access, Watch, and Reserve Adoption. Antibiotics.

[8] Biro A., Elek P. (2019). The effect of primary care availability on antibiotic consumption in Hungary: A population based panel study using unfilled general practices. BMJ Open.

[9] García P.R., Villar F.A. (2019). Effects of economic and health policies on the consumption of antibiotics in a Spanish region. Expert Rev. Pharmacoeconomics Outcomes Res..

[10] Rojas P., Antoñanzas F. (2020). Policies to Reduce Antibiotic Consumption: The Impact in the Basque Country. Antibiotics.

[11] Nga D.T.T., Chuc N.T.K., Hoa N.P., Nguyen N.T.T., Loan H.T., Toan T.K., Phuc H.D., Horby P., Van Yen N., Van Kinh N. (2014). Antibiotic sales in rural and urban pharmacies in northern Vietnam: An observational study. BMC Pharmacol. Toxicol..

[12] Costantini S., Walensky R.P. (2019). The Costs of Drugs in Infectious Diseases: Branded, Generics, and Why We Should Care. J. Infect. Dis..

[13] Pulcini C., Beovic B., Béraud G., Carlet J., Cars O., Howard P., Levy-Hara G., Li G., Nathwani D., Roblot F. (2017). Ensuring universal access to old antibiotics: A critical but neglected priority. Clin. Microbiol. Infect..

[14] Nass S.J., Madhavan G., Augustine N.R. (2017). Making Medicines Affordable: A National Imperative. National Academies of Sciences, Engineering, and Medicine, Health and Medicine Division, Board on Health Care Services, Committee on Ensuring Patient Access to Affordable Drug Therapies.

[15] (2008). Measuring Medicine Prices, Availability, Affordability and Price Components.

[16] Klein E.Y., Milkowska-Shibata M., Tseng K.K., Sharland M., Gandra S., Pulcini C., Laxminarayan R. (2020). Assessment of WHO antibiotic consumption and access targets in 76 countries, 2000–15: An analysis of pharmaceutical sales data. Lancet Infect. Dis..

[17] Versporten A., Zarb P., Caniaux I., Gros M.-F., Drapier N., Miller M., Jarlier V., Nathwani D., Goossens H., Koraqi A. (2018). Antimicrobial consumption and resistance in adult hospital inpatients in 53 countries: Results of an internet-based global point prevalence survey. Lancet Glob..

[18] Abu-Ajaleh S., Elhajji F.D., Al-Bsoul S., Abu Farha R., Al-Hammouri F., Amer A., Al Rusasi A., Al-Azzam S., Araydah M., Aldeyab M.A. (2023). An Evaluation of the Impact of Increasing the Awareness of the WHO Access, Watch, and Reserve (AWaRe) Antibiotics Classification on Knowledge, Attitudes, and Hospital Antibiotic Prescribing Practices. Antibiotics.

[19] Al-Azzam S., Mhaidat N.M., Banat H.A., Alfaour M., Ahmad D.S., Muller A., Al-Nuseirat A., Lattyak E.A., Conway B.R., Aldeyab M.A. (2021). An Assessment of the Impact of Coronavirus Disease (COVID-19) Pandemic on National Antimicrobial Consumption in Jordan. Antibiotics.

[20] Elhajji F.D., Al-Taani G.M., Anani L., Al-Masri S., Abdalaziz H., Qabba’h S.H., Al Bawab A.Q., Scott M., Farren D., Gilmore F. (2018). Comparative point prevalence survey of antimicrobial consumption between a hospital in Northern Ireland and a hospital in Jordan. BMC Heal. Serv. Res..

[21] Abu Hammour K., Al-Heyari E., Allan A., Versporten A., Goossens H., Abu Hammour G., Manaseer Q. (2020). Antimicrobial Consumption and Resistance in a Tertiary Care Hospital in Jordan: Results of an Internet-Based Global Point Prevalence Survey. Antibiotics.

[22] World Health Organization World Health Organization Model List of Essential Medicines—23rd List, 2023. Proceedings of the Selection and Use of Essential Medicines 2023: Executive Summary of the Report of the 24th WHO Expert Committee on the Selection and Use of Essential Medicines.

[23] WHO Collaborating Centre for Drug Statistics Methodology and Norwegian Institute of Public Health (2023). ATC/DDD Index 2023. Oslo. https://www.whocc.no/atc_ddd_index/.

[24] Jordan Food and Drug Administration Drug information, Prices, and Leaflets. http://www.jfda.jo/Pages/viewpage.aspx?pageID=184.

[25] WHO Collaborating Centre for Drug Statistics Methodology and Norwegian Institute of Public Health (2023). DDD Definition and General Considerations. Oslo. https://www.whocc.no/ddd/definition_and_general_considera/.

[26] Gauthaman J., Jayanthi M. (2022). Wide Cost Variations Observed in Antibiotics and Analgesics Prescribed for Dental Care in India: A Price and Affordability Analysis. Cureus.

[27] Jordanian Ministry of Labor The Tertiary Labor Committee Keeps the Minimum Wage Limit. February 2023. https://mol.gov.jo/ar/NewsDetails/%d8%a7%d9%84%d9%84%d8%ac%d9%86%d8%a9_%d8%a7%d9%84%d8%ab%d9%84%d8%a7%d8%ab%d9%8a%d8%a9_%d9%84%d8%b4%d8%a4%d9%88%d9%86_%d8%a7%d9%84%d8%b9%d9%85%d9%84_%d8%aa%d8%a8%d9%82%d9%8a_%d8%b9%d9%84%d9%89_%d8%a7%d9%84%d8%ad%d8%af_%d8%a7%d9%84%d8%a3%d8%af%d9%86%d9%89_%d9%84%d9%84%d8%a3%d8%ac%d9%88%d8%b1.

